# Proposed New Hospitals upon Randall’s Island, New York

**Published:** 1867-02

**Authors:** 


PROPOSED NEW HOSPITALS UPON RANDALL'S ISLAND, NEW YORK.—At a semi-monthly meeting, in December last, of the Commissioners of Public Charities and Correction, it was decided to establish a hospital for incurables at the alms-house on Randall's Island. The architect of the Board also received orders to prepare a design for an insane hospital capable of accommodating eighty persons.

